# Fall Prevention Self-Assessments Via Mobile 3D Visualization Technologies: Community Dwelling Older Adults’ Perceptions of Opportunities and Challenges

**DOI:** 10.2196/humanfactors.7161

**Published:** 2017-06-19

**Authors:** Julian Hamm, Arthur Money, Anita Atwal

**Affiliations:** ^1^ Department of Computer Science Brunel University London United Kingdom; ^2^ School of Health and Social Care London South Bank University LONDON United Kingdom

**Keywords:** health informatics, falls, occupational therapy, assistive equipment provision process, self-assessment, measurement guidance, extrinsic risk factors, 3D visualization, technology-based systems

## Abstract

**Background:**

In the field of occupational therapy, the assistive equipment provision process (AEPP) is a prominent preventive strategy used to promote independent living and to identify and alleviate fall risk factors via the provision of assistive equipment within the home environment. Current practice involves the use of paper-based forms that include 2D measurement guidance diagrams that aim to communicate the precise points and dimensions that must be measured in order to make AEPP assessments. There are, however, issues such as “poor fit” of equipment due to inaccurate measurements taken and recorded, resulting in more than 50% of equipment installed within the home being abandoned by patients. This paper presents a novel 3D measurement aid prototype (3D-MAP) that provides enhanced measurement and assessment guidance to patients via the use of 3D visualization technologies.

**Objective:**

The purpose of this study was to explore the perceptions of older adults with regard to the barriers and opportunities of using the 3D-MAP application as a tool that enables patient self-delivery of the AEPP.

**Methods:**

Thirty-three community-dwelling older adults participated in interactive sessions with a bespoke 3D-MAP application utilizing the retrospective think-aloud protocol and semistructured focus group discussions. The system usability scale (SUS) questionnaire was used to evaluate the application’s usability. Thematic template analysis was carried out on the SUS item discussions, think-aloud, and semistructured focus group data.

**Results:**

The quantitative SUS results revealed that the application may be described as having “marginal-high” and “good” levels of usability, along with strong agreement with items relating to the usability (*P*=.004) and learnability (*P*<.001) of the application. Four high-level themes emerged from think-aloud and focus groups discussions: (1) perceived usefulness (PU), (2) perceived ease of use (PEOU), (3) application use (AU) and (4) self-assessment (SA). The application was seen as a useful tool to enhance visualization of measurement guidance and also to promote independent living, ownership of care, and potentially reduce waiting times. Several design and functionality recommendations emerged from the study, such as a need to manipulate the view and position of the 3D furniture models, and a need for clearer visual prompts and alternative keyboard interface for measurement entry.

**Conclusions:**

Participants perceived the 3D-MAP application as a useful tool that has the potential to make significant improvements to the AEPP, not only in terms of accuracy of measurement, but also by potentially enabling older adult patients to carry out the data collection element of the AEPP themselves. Further research is needed to further adapt the 3D-MAP application in line with the study outcomes and to establish its clinical utility with regards to effectiveness, efficiency, accuracy, and reliability of measurements that are recorded using the application and to compare it with 2D measurement guidance leaflets.

## Introduction

### Fall Prevention Technologies and Patient Involvement

Due to an ageing world population, the number of fall-related injuries has risen in recent years, hence, posing a significant global health challenge [1]. Approximately 30% of older adults over 65 years and 50% of adults over 80 years who live independently fall each year [2]. Falls result in injuries that can precipitate early hospital and long-term care admissions, and, in some cases, can be the cause of death. The result is an ever increasing demand for health and social care services and resources [3,4]. In the United Kingdom, the cost of falls to the National Health Service (NHS) is estimated at over £2.3 billion per year. Government and health authorities see new and innovative applications of information and communication technologies (ICTs) within the falls prevention domain as having the potential to reduce health care costs while also addressing the increased burden that an ageing population places on health and social care services [5]. In particular, ICTs deployed within the health sector are seen as having the potential to enable patients to self-assess, self-manage, and provide self-care, thus reducing the demand for clinicians in the delivery of a range of health interventions [6,7]. Additional anticipated benefits of technology-assisted interventions include a potential rise in levels of patient engagement and adherence, which may in turn result in higher levels of overall patient satisfaction and quality of life [5]. Already, there appears to be a shift away from the traditional paternalistic models of health care where the patient is a passive recipient toward more patient-centered models where the patient is given more responsibility for providing their own care such as carrying out self-assessments and management of their own conditions [8]. Part of this change is related to the emergence of the notion of the “expert patient,” one who is expected to be able to access relevant information, utilize self-testing and manage medical devices and applications effectively, and make independent decisions about their own care [9,10].

In the field of occupational therapy, the assistive equipment provision process (AEPP) is widely used as a prevention strategy to promote independent living, and to identify and mitigate falls risk factors via the provision of assistive equipment (also referred to as assistive technology) where appropriate. AEPP involves occupational therapists (OTs) working, often with older adult patients, to identify intrinsic and extrinsic falls risk factors that impact patients’ ability to carry out activities of daily living (ADL). Intrinsic risk factors include functional ability deficits and cognitive and balance impairments. Extrinsic risk factors include poor lighting, slippery surfaces, and obstacle and trip hazards as well as inappropriate or “poor fit” of assistive equipment and lack of stair handrails and bathroom grab rails [11]. Existing research literature indicates that much effort has been focused on developing technology-based systems and software applications that attempt to mitigate intrinsic fall risks [12,13], however, comparatively little effort has been invested into developing technology-based systems that address and overcome extrinsic risk factors.

### Assistive Equipment Provision and Patient-Led Self-Assessment

The aim of the AEPP is to reduce barriers that impact patients’ ability to perform day-to-day living tasks and reduce fall risk factors. This is typically achieved by recommending adaptations to the home and the installation of assistive equipment such as bath boards, shower chairs, toilet raisers, chair raisers, bed raisers, and grab rails to help with transfers when bathing or climbing stairs [14]. Adaptation of the home and installation of such equipment is carried out to accommodate functional changes, assist with ageing-in-place, and reduce fall risk factors [15]. A key strategy to mitigate the adverse impact of functional decline is to identify and accurately prescribe assistive equipment that will sustain independent living and quality of life [15-18]. Therefore, clinicians undertake home visits to assess functional abilities and take measurements from the patient, fittings, and key items of furniture that form the basis upon which assistive equipment and home adaptations are prescribed. Recorded measurements inform the precise type, size, and nature of the assistive equipment that is prescribed, and therefore, play a vital role in ensuring the successful fit between the assistive device and the person using it [19,20].

**Figure 1:**
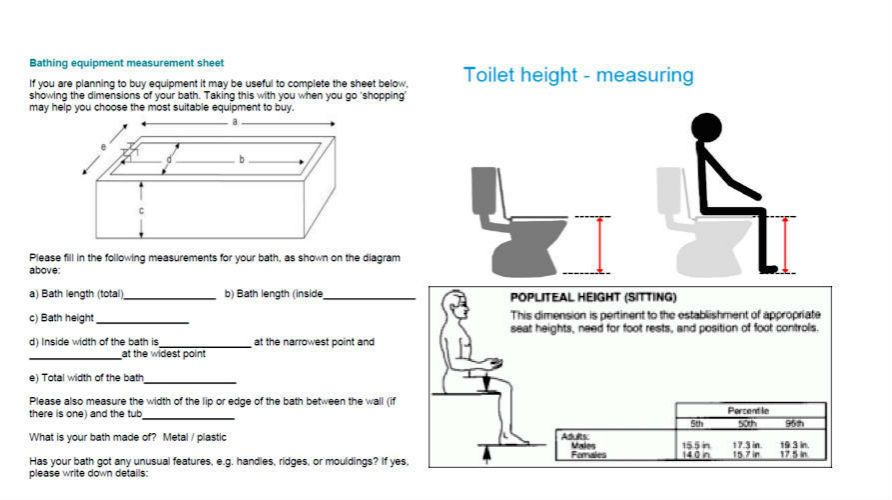
2D paper-based measurement guidance form used within the AEPP in practice. AEPP: assistive equipment provision process.

Currently, paper-based forms are used in the AEPP to record measurements and associated patient data. These forms include measurement guidance that is presented in the form of 2D illustrations of information that must be collected from key items of home furniture, fittings, and the patient. The paper-based 2D illustrations are typically annotated with measurement arrows that serve as prompts to indicate the precise points in 3D space that must be accurately identified and measured by the clinician in order to gather the necessary data to formulate an assessment; the data is subsequently used to prescribe the necessary home adaptations and assistive equipment [21]. Figure 1 shows some examples of paper-based forms and the 2D illustrations that are currently used as part of the AEPP [22,23].

Despite the importance of accurate measurement and the prominent use of 2D paper-based measurement guidance, approximately 50% of assistive equipment that is prescribed as a result of the AEPP is abandoned by service users [24]. One of the principal reasons of equipment abandonment is due to “poor fit” between the equipment and the individual using it, despite the fact that trained OTs carry out the measurement tasks [24,25]. The impact of this “poor fit” issue is significant and wide-spread and negatively impacts patient health outcomes, accelerates functional decline, increases overall exposure to fall risks in the home, and more generally, unnecessarily depletes already scarce and valuable health care resources [26,27]. To compound this issue, it is anticipated that due to time and OT resource limitations, the responsibility of taking and recording of measurements will soon become that of the service users or their family members or carers [28]. Given the issues of “poor fit” that already arise as a result of trained OTs carrying out these tasks, it is likely that “poor fit” will remain a significant issue if patients and carers will be given the responsibility for carrying out these skilled tasks [29]. Indeed, patients taking their own measurements and carrying out self-assessment has already become part of practice in some NHS trusts in the United Kingdom [28]. However, little is known with regards to the tools that patients use to facilitate taking and recording accurate measurements as part self-assessing for equipment to ensure successful or correct fit of equipment [30], particularly given the patients desire to being involved in self-assessing for the provision of equipment. If patients, family members, and carers are to be able to carry out the AEPP effectively, there is a need to be supported via the provision of appropriate information, training, and new and innovative tools that provide clear and effective guidance, support, and facilitate the necessary gathering of reliable and accurate information. Currently, there appears to be no such tool or guidance designed specifically for use by patients to carry out the AEPP.

### 3D Visualization Technologies for Guiding Assistive Equipment Provision Process (AEPP) Self-Assessment

The term 3D visualization refers to computer graphics software applications that capitalize upon natural aspects of human perception by visually simulating 3 spatial dimensions in 2D space, hence enabling the user to visualize, interact with, and control a given object within a 3D space. The value of 3D visualization technologies in the falls prevention research domain has already been demonstrated in a number of existing studies that focus on the areas of exercise intervention. Some examples include Uzor et al [31] and Doyle et al [32] who aim to improve uptake and adherence to home-based falls prevention exercise programs by replacing traditional paper-based 2D illustrated exercises with equivalent interactive 3D visualization of these programs. One existing study explores the potential of exploiting 3D visualization technologies to assist clinicians in identifying extrinsic fall hazards. Du et al [33] developed a robotic system to automatically model patients’ home environments in 3D space. A 3D visualization of the environment is constructed, with the help of the robot, to assist clinicians in identifying the precise location and nature of extrinsic fall hazards. Examples in other areas of health care include the work of Spyridonis et al [34] who found that enabling patients to carry out self-assessments by reporting the type and precise location of back pain by using a 3D visualization of the human body was more accurate and intuitive than the traditional paper-based 2D model of the human body typically used in practice. Other studies have found similar benefits in utilizing 3D visualizations to communicate other forms of pain to clinicians. For example, Jang et al [35] enable patients to express and communicate their symptoms of pain to clinicians by annotating specific regions on an on-screen 3D representation of the human body using free-hand drawing. De Heras Ciechomski et al [36] propose a preoperative surgical 3D visualization system for breast augmentation using 2D digital photographs of the patient’s torso and reconstructing these into 3D models. This system helps clinicians to perform virtual clinical analysis without the patient being present and visualizes the required measurements on the modeled body in order to facilitate accurate measurements for the treatment.

The research literature to date indicates that the use of 3D visualizations have shown promise in providing opportunities to overcome the challenges of existing 2D clinical tools to sufficiently provide the visual quality necessary to conceptualize visual cues as part of a particular treatment and assessment [37,38]. In light of the equipment abandonment issues faced by the current AEPP process, discussed previously, there is a need to explore the potential value of 3D visualization applications developed specifically for use by service users that serve as an aid in the process of carrying out the key measurement tasks that form part of the AEPP.

### Patient Perceptions and Acceptance of Technology

The effective design of health technologies that are usable and deliver functionality aligned with the needs and preferences of the patient is as important as the innovation itself [39], since this is likely to realize higher levels of engagement and adoption of a given technological innovation [40-42]. Consequently, it is vital that patient experiences and perceptions are sought and explored if new tools and technologies are to be viable, accepted, and usable in clinical practice [43]. Involving end users in the development of technology in a formalized manner ensures that user needs, design considerations, and crucial aspects of clinical interventions are appropriated within the design and development process. A number of formal user-centered methods [39] and technology adoption theories are available to gain valuable insights into user needs and perceptions of technology, and they can be factored into the design of that technology [44,45]. For example, the technology acceptance model (TAM) has been increasingly seen within the health care field as an appropriate theory used to better understand factors that predict actual system use, adoption, and acceptance [46,47]. Recent research has explored both clinicians and patients’ responses using TAM within a quantitative context [48]; however, the use of TAM in qualitative work has become increasingly recognized [46]. More specifically, to use the predefined high-level TAM constructs such as perceived usefulness (PU) and perceived ease of use (PEOU) as a deductive framework by means of which user perceptions of emerging technologies may be interpreted and made sense of [49]. In turn, the perceptions of users may be used to inform the iterative design and development of proposed technological innovations within a health care context.

This study presents a novel mobile 3D visualization application prototype designed to provide measurement guidance to users as part of the AEPP. The aim of this study was to investigate the perceptions of community dwelling older adults regarding the feasibility, benefits, and challenges of using a 3D visualization technology application to facilitate carrying out AEPP self-assessment tasks in practice. The next section describes the initial design phase activities and provides a detailed walkthrough of the application prototype and system architecture. Next, the main study is presented along with the methods used to explore the experiences and views of community-dwelling older adults after using the 3D visualization application for carrying out AEPP measurement tasks. The results of the main study are then presented followed by a discussion of the findings and implications and recommendations for use of the 3D visualization application in practice. Conclusions are then drawn, along with details of future research directions.

### Concept Design Phase and Application Walkthrough

This section provides details of the initial concept design phase and a walkthrough of the prototype application developed for use in the main study.

#### Initial Concept Design of the Prototype Application

**Figure 2:**
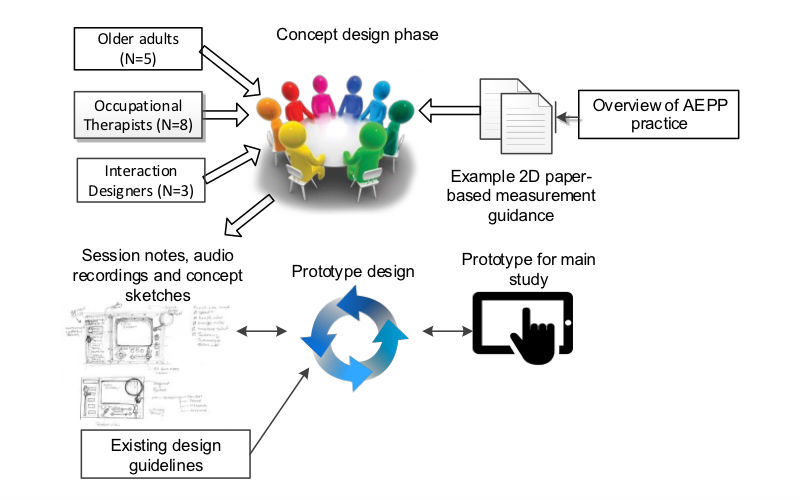
Overview of the procedure for the initial concept design phase.

In order to significantly improve user experience, usability evaluations should be performed continuously through the early stages of low and high fidelity prototype development [50]. Therefore, an initial concept design phase was undertaken to inform the overall design and development of the 3D measurement aid prototype (3D-MAP). User-centered design methods and design guidelines were employed and adhered to in this phase to ensure the application was aligned with the needs of the intended users [51,52]. Figure 2 presents an overview of the protocol followed during this phase.

A total of 3 interaction designers, 5 community-dwelling older adults, and 8 OTs took part in the concept design phase. The objectives of the session were to identify high-level requirements specific to the application and to reflect on existing evidence-based practice and explore ways in which the application could be designed to support current practice. An overview of existing AEPP practice was presented to participants at the start of the session. In addition, samples of existing 2D paper-based measurement guidance leaflets were provided as a point of reference to encourage older adults to design their sketches in accordance with the provided materials. Participants were also shown examples of existing clinical visualization applications, which demonstrated how 2D illustrations may be presented using 3D visualization technologies on mobile phones, tablets, and laptops.

**Figure 3:**
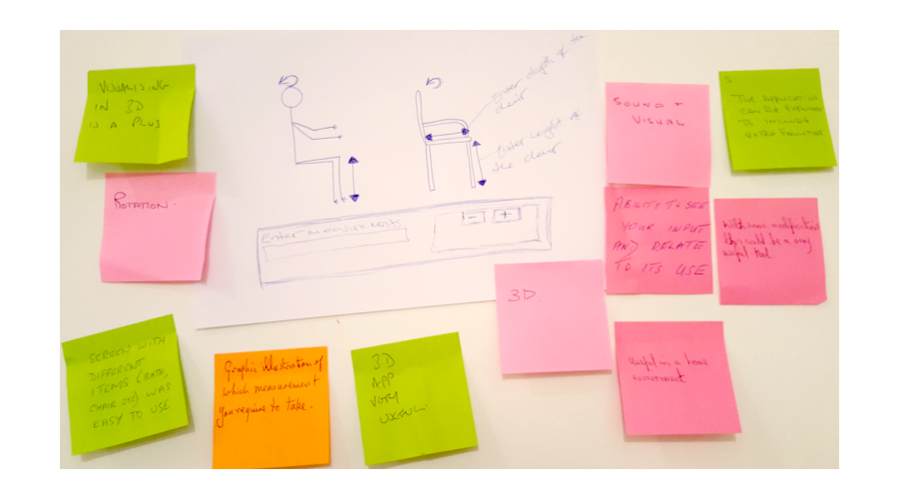
Concept sketches of a self-assessment tool designed by older adults during the participatory design sessions.

Participants were asked to explore the idea of utilizing a software application to enable AEPP self-assessment tasks and to suggest key design features and functionality that not only matched but enhanced the conventional 2D leaflets if the application were to replace them. As a result, participants were encouraged to sketch out rough ideas, and with help from interaction designers, fine-tune these ideas into more complete annotated concept sketches of a potential application interfaces and associated functionality. Figure 3 presents an example of a concept sketch produced during the initial concept design session.

Once all participatory design sessions were completed, notes and recordings of the sessions along with the annotated concept sketches were perused and used to inform the design and development of the 3D-MAP application. A total of 8 user requirements (UR1-UR8) were identified as a result of this concept design phase.

OTs believed that measurements should be recorded electronically to remove the need to keep paper records of measurements (UR1). They envisaged the application would allow them to annotate 3D representations of the item by adding measurements directly to the item being modeled by the application. UR2 reflects the fact that OTs stated they required a clean looking interface that includes only necessary information or functionality to enable them to carry out the task at hand. Enabling the user to rotate and zoom the position and the view perspective of the 3D model to improve interpretation of clinically significant landmarks was also seen as crucially important (UR3). Participants suggested that arrows (as often used in 2D paper-based guidance) would serve as a useful prompt to provide guidance but could also serve as a feature for inputting measurements when clicked (UR4). Automatic generation of assessment reports, as much of what the application provides, was seen as a potential feature (UR5). OTs also saw potential for integrating data collected automatically into patient records (UR6). Given the nature of carrying out home visit assessments often in patient’s homes, it was more fitting for the application to be deployed on a mobile platform, given the reported increase of use of mobile devices within OTs professional roles (UR7). Clinicians felt that audio instructions that guide the user while using the application would be useful for them but also for patients if they were ever expected to use such an application (UR8).

### Overview of the 3D Measurement Aid Prototype (3D-MAP) Prototype

The 3D-MAP application, which is an interactive and functional application (high fidelity prototype) utilizing 3D visualization technology, has been developed according to the user requirements and concept sketches that emerged from the initial concept design phase, and it is consistent with existing 3D visualization guidelines presented in the existing research literature [53-56]. The system design and architecture of 3D-MAP is now presented along with an application walkthrough.

#### System Architecture

**Figure 4:**
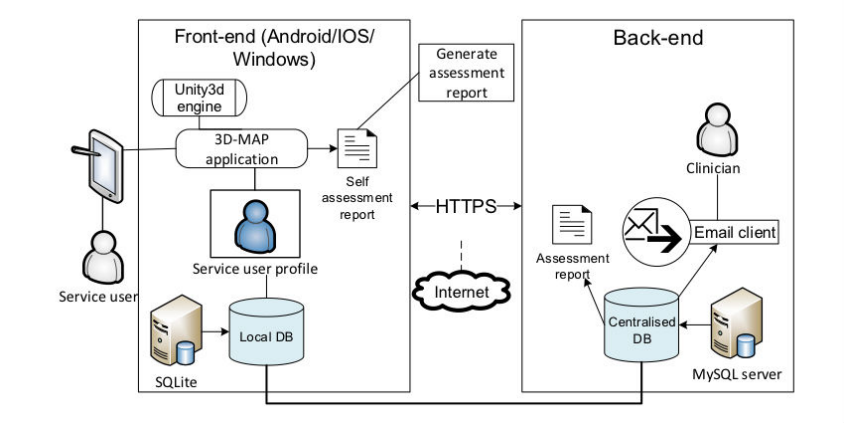
3D measurement aid prototype (3D-MAP) system architecture.

The deployment platform for the 3D-MAP prototype was the Android operating system (OS), which is an open-source platform that is freely available for both personal and commercial use. In the anticipation of the prototype being used on multiple platforms, the prototype was developed using the Unity3D game engine that allows applications to be deployed on multiple platforms including Android, iOS, and Windows (UR7). Unity3D (Unity Technologies, San Francisco, United states) is a tool chosen for its capabilities of rending 3D models and deploying applications on mobile devices seamlessly. Considering the user requirements that emerged from the initial concept design phase, Figure 4 shows the underlying system architecture of the 3D-MAP prototype.

The user has the ability to input measurements by using the device’s touchscreen. Measurements are stored in a local database located on the device. The stored data is then transmitted through hypertext transfer protocol secure (HTTPS) to a centralized MySQL database in encrypted format and is only accessible by authorized clinical users. All relevant application data stored locally is encrypted and is deleted from the device subsequent to sending the data to the centralized database. The users have the option of generating a self-assessment report (UR5). Once the necessary data is collected, the user can email the assessment report to their appointed clinician. Data collected are both stored locally and remotely to a service user profile. This offers a workable electronic record for each patient that has received assessments from clinicians and for those who have self-assessed; this application also offers capabilities of sharing patient records to other clinicians.

#### Application Walkthrough

**Figure 5:**
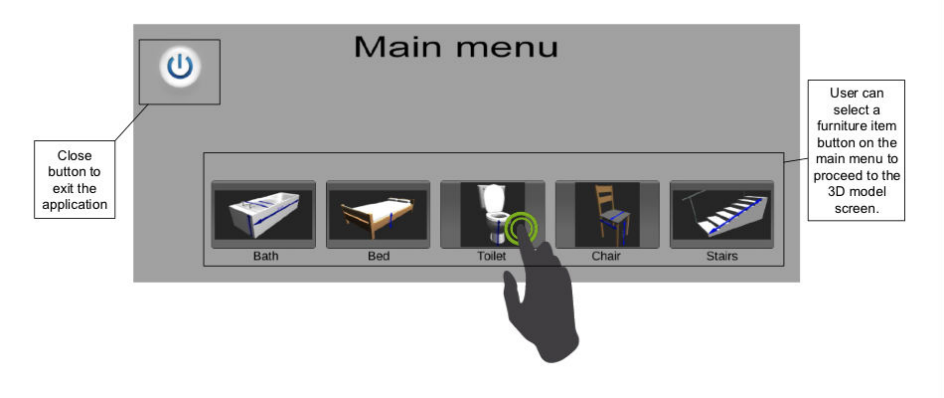
3D measurement aid prototype (3D-MAP) application (main menu).

A crucial feature of the application is the audio prompt and visualization of the measurement guidance. The application displays 3D models of the 5 home furniture items (bed, bath, toilet, chair, and stairs), that are most commonly associated with extrinsic falls within the home environment [24] and are therefore typically measured as part of the AEPP. Arrows are used as prompts to indicate discrete points on the home furniture to be measured (UR4). The 3D models and measurement arrows were developed using Blender (Stichting Blender Foundation, Amsterdam), which is an open-source software package for designing 3D graphics and animation [57]. The models were then converted into an “.obj” file format and imported into Unity3D. More specifically, the measurement guidance was presented using two prompt features: arrows and audio prompt (UR8). Measurement guidance is available for each respective furniture item from the main menu as shown in Figure 5 (UR2).

**Figure 6:**
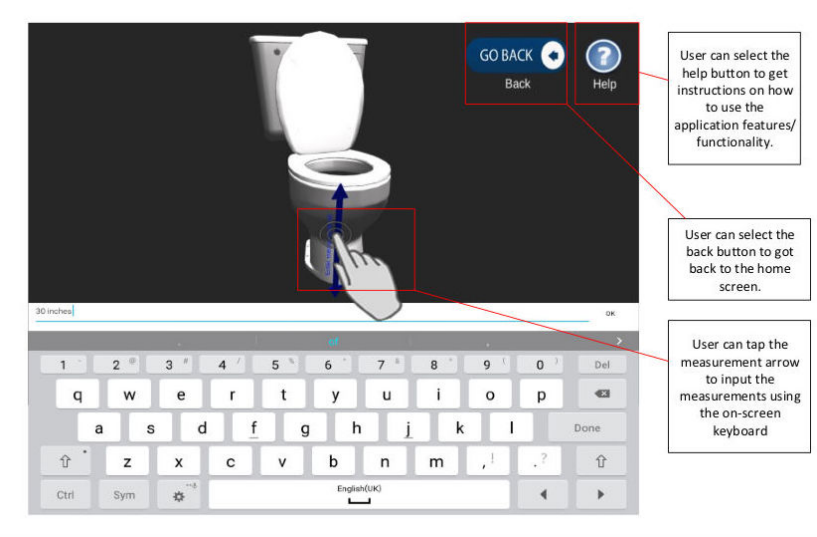
3D toilet model screen.

The user is presented with the home furniture item interface and with the necessary measurement prompts, using indicative arrows superimposed onto each respective 3D model within 3D space. These measurement prompts were positioned as indicated by existing 2D paper-based guides, the positions of which were verified via careful consultation with clinicians who specialize in the AEPP measurement training. An example of the toilet scene, including a measurement prompt is given in Figure 6.

The 3D models of the items of furniture and prompts (arrows) were developed using Blender [57], converted into an “.obj” file format and imported into Unity3D. The measurement guidance is presented using two prompt features: 3D arrow lines and audio instructions that guide the user to provide the necessary measurements. In line with the requirements gathered from the initial concept stage, written instructions from the paper-based forms were taken and translated into audio files. Audio cues (UR8) are activated when the arrows are touched providing instruction on how and where to accurately measure specific parts of home furniture (UR4).

**Figure 7:**
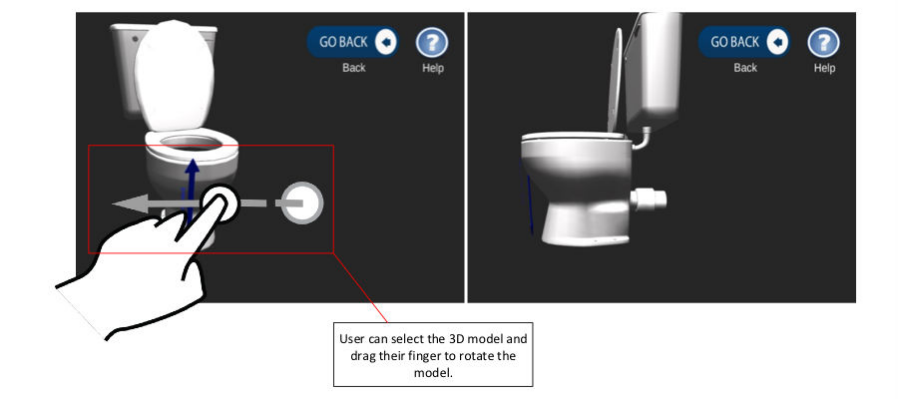
Rotation feature to manipulate 3D models to facilitate gaining a better understanding of the clinical guidance.

Users have the ability to rotate the 3D furniture models to view discreet areas of interest in detail (UR3). In order to do so, the figure swipe gesture input was employed, which enabled the handling of rotating the models. Figure 7 presents an example of rotating one of the models clockwise, by the user swiping their finger horizontally to the left in order for the model to follow suit, similarly, a horizontal swipe to the right rotates the model anticlockwise.

**Figure 8:**
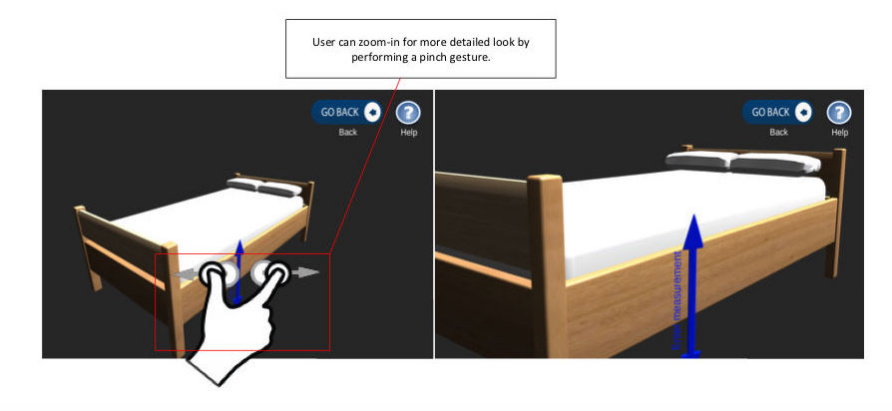
Zoom in/out feature to manipulate 3D models to facilitate gaining a better understanding of the clinical guidance.

Another key component of the design is the zoom-in and zoom-out feature (UR3), which changes the viewpoint and perspective for a more detailed look at the 3D furniture models by using the pinch gesture to achieve this (as shown in Figure 8).

The application enables users to input home furniture measurements via the use of the arrow prompts augmented with sound instructions (UR8). The application is flexible in relation to the interface used, the visualization capability and audio cue options provided to users are also optional for those who feel they have grasped the use of the application and no longer require audio assistance.

## Methods

### Overview

**Figure 9:**
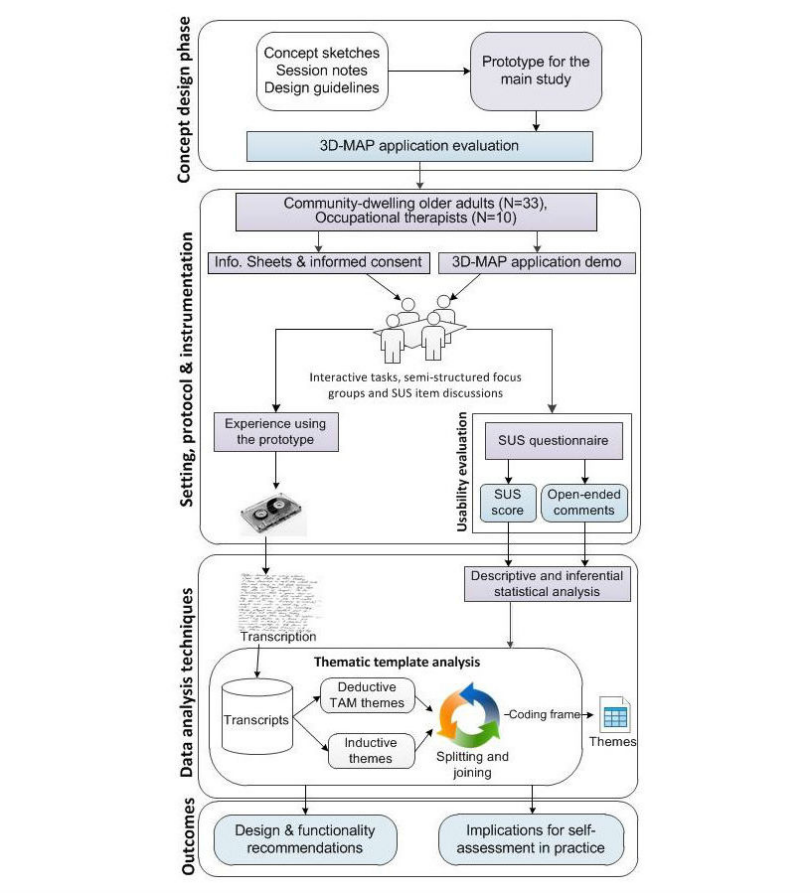
Overview of the session, methods, and process.

This section provides details of the data collection and analysis methods used to explore the perceptions of community-dwelling older adults regarding the use of the 3D-MAP application as a self-assessment tool within AEPP in practice. Figure 9 presents an overview of the study design, methods, and research instruments employed to produce study outcomes and recommendations for practice.

### Participants

A purposive sampling strategy was used for recruitment of participants for this study, for which a total of 33 community-dwelling older adults were recruited. This was in line with a posthoc power analysis that was performed, which indicated that a similar sample size of 33 participants was sufficient (power=0.80) to detect a large effect (0.5) with alpha set at .05, 2-tailed. Participants were recruited through a number of different sources. In the first instance, managers of leisure centers that run exercise classes for 50+ groups were contacted as gatekeepers to disseminate invitations to older adults. A total of 18 participants were recruited through the “active 50’s” group at Brunel University and 15 participants through the “active lifestyles” group in the area of South West London. A financial incentive of a £10 voucher was offered in acknowledgement of participants who agreed to take part. The inclusion criteria for selection were that participants were over the age of 50 years, familiar with or had basic skills of using technology (eg, the use of desktop computers, laptops, mobile phones), and considered themselves as active and healthy. Each participant reported their familiarity with touchscreen technology used within their personal and in some cases professional lives. Twenty-three of the participants were female (70%, 23/33) and 10 (30%, 10/33) were male; (23 females; 10 males, mean age=71.2 years, range=56-89, standard deviation=8.3). The majority of participants were retired or semiretired with the exception of 2 who were in full-time employment. This sample had no prior exposure to using self-assessment tools for the AEPP, however, 5 participants reported to have second-hand experience of family members having their home adapted due to ageing changes. Table 1 provides the demographics and summary of participant profiles for this study.

**Table 1:** Summary of participant profiles.

Part. ID	Gender	Age in years; mean (SD), range	Occupation	Group number
#F1-#F8	2 F^a^, M^b^, 5 F	66.2 (7.7), 52-75	6-retired, aircrew, flight manager, administration	1
#F9-#F14	M, 2 F, 3 M	75.2 (7.9), 65-86	Retired	2
#F15-#F18	M, F, M, F	71.0 (3.7), 66-75	Retired	3
#F19-#F27	M, F, M, 2 F, M, 3 F	70.6 (9.6), 54-89	Retired	4
#F28-#F33	6 F	76.2 (6.4), 68-87	Retired	5

^a^F: female.

^b^M: male.

### Protocol and Instrumentation

Participant sessions were conducted on a one-to-one basis for the main interaction task, followed by a series of focus group sessions to discuss participant experiences of using the 3D-MAP application. The total duration of each session were approximately 90 min. Each session consisted of five key stages: (1) issue information sheet, question and answer, and complete consent form (individual); (2) provide a demonstration of the 3D-MAP application and answer questions (individual); (3) carry out the interactive task using the 3D-MAP application (individual); (4) administer system usability scale (SUS) questionnaire and retrospective think-aloud discussion (individual); and (5) follow-up focus group discussions about individual SUS items and perceptions and experiences of using the application (group).

An information sheet was given to participants on arrival before taking part in the session; this provided a background, aim of the study, and listed tasks that participants were expected to perform during the session. The content was worked through with each participant. They were continuously given the opportunity to ask questions to resolve any misunderstandings or queries. Informed consent was obtained by asking participants to complete a consent form, which explained their ethical rights to withdraw from the study at any time without having to provide any reason. Participants were given a brief demonstration of the 3D-MAP application, which included showcasing key features of the application, inputting measurements, and generating assessment reports. At this point, further information was provided regarding the application and participants were allowed to practice using it, while being individually supervised by a facilitator who answered any questions as they arose. The participants were allowed to provide their thoughts and feedback on their first impression of the application during the demonstration. Subsequently, participants were then set up with the application on their Android tablet and were asked to use the application, and were given written instructions outlining a series of tasks to perform using the application. Textbox 1 presents the key steps involved in interacting with the 3D-MAP application.

**Written instructions for the interactive task.**

Instruction sheet for participants

- Start the application
- Select a home furniture from the main menu screen
- Rotate 3D model left or right and up or down
- Zoom in and out using the pinch touch gesture
- Click on arrows to activate the audio prompt
- Measure the 5 home furniture items
- Enter measurements using the virtual popup keyboard
- Click on the main menu button (move on to the next furniture item)

For the interactive task, participants were asked to use the application and to manipulate the viewpoints or position of the 3D furniture models to obtain the necessary depth of clinical guidance to measure the 5 home furniture items. Participants were encouraged to verbalize their thoughts immediately after interacting with the application, while adopting a retrospective think-aloud approach (otherwise known as think-after [58]) immediately after interacting with the application [59]. This provided insights into the usability of the application, thus resulting in additional qualitative data. The think-aloud approach is a well-established technique used for gathering thoughts of users while they are interacting with a software application. The technique is particularly useful to gain insights and understand the reasoning behind participants’ preferences and thoughts. It is most commonly used in usability testing studies and has been employed to study older adults’ interactions with user interfaces (UIs) and ways in which they structure their tasks when using the interface [60]. Variants of the technique such as concurrent think-aloud has limitations when being used with this user cohort, particularly those who exhibit cognitive impairments, find unfamiliar interfaces challenging to use, and employing the technique can hinder the completion of the task [60,61]. With this in mind, retrospective think-aloud was, therefore, adopted to get participants to explain their behavior after completing the tasks [58]. Users were reassured that there was no urgency in completing the task and were encouraged to take as long as they felt necessary to verbalize their thoughts while interacting with the application. Think-aloud prompts such as “what did you think at this moment?” and “what were you thinking?” were used after completing the task and whenever there were long periods of silence [62]. Furthermore, participants were encouraged to use the application to stimulate think-after thoughts. Participants were asked to complete a SUS questionnaire [63] on completion of the interaction task, which was used to evaluate the general usability of the 3D-MAP prototype. SUS is a 10-item questionnaire instrument that asks users to rate a system against a list of items on a 5-point Likert scale from 1=“strongly disagree” to 5=“strongly agree.” The word “cumbersome” in SUS item 8: “I found the system to be very cumbersome to use” was replaced with “awkward” to increase comprehension as suggested by Bangor et al [64]. Each SUS item was further modified by replacing “system” with “3D-MAP application” to assist users in scoring the application accurately. Such changes to SUS are standard practice and have no impact on the questionnaire’s validity or reliability [65]. The SUS produces a score that represents a quantitative measure of the general usability of a system (for this study 3D-MAP application). After completion of the SUS instrument, participants were asked to discuss the score they attributed to each respective SUS item. Focus groups were conducted in a semistructured format with participants who were asked to discuss their experience of using the application with respect to each individual SUS statement, and then more generally about their perceptions of the opportunities and challenges of the 3D-MAP prototype as a self-assessment tool in practice. In total, 5 focus groups were undertaken and the number of participants in each group varied (n=8, 6, 4, 9, and 6, respectively). The number of focus groups and sample of participants in each group is in line with the minimum 4 focus group rule and the recommended 4-12 participant threshold [66] that is considered to be suitable numbers for conducing focus groups within a health care context [67]. Written notes were being taken by moderators to supplement the analysis of later discussions held at the end of the sessions.

### Data Analysis

IBM SPSS statistical software package version 20.0.0 was used to analyze the SUS responses collected for this study. The quantitative data collected in this study was subjected to descriptive and inferential statistical analysis. To better understand and interpret the SUS scores, the adjective [68] and curved grading scales [69,70] were used to analyze and interpret the SUS scores. This involved calculating a SUS score from the completed questionnaires, and generating a value on a 100-point rating scale, which may then be mapped to descriptive adjectives (best imaginable, excellent, good, OK, poor, and worst imaginable), an acceptability range (acceptable, marginal-high, marginal-low, and not acceptable), and a curved grading sale (F=absolutely unsatisfactory to A+=absolutely satisfactory) *. These baseline ranges and grading are derived from* a sample of over 3000 software applications that provide the comparative baseline [68]. Until recently practitioners viewed SUS as unidimensional until Lewis and Sauro [65] concurrently with Borsci et al [71] proposed SUS is composed of a two-factor structure in which 2 subscales, namely, usability (SUS items S1, S2, S3, S5, S6, S7, S8, and S9) and learnability (SUS items S4 and S10) underpin the SUS instrument. Additional statistical analysis was performed using one-sample *t*-test to establish whether there were significant differences between the respective mean SUS scores and the midpoint value of three (of the 5-point Likert type scale responses) for each individual SUS item and for the usability and learnability constructs.

Audio recordings of SUS item discussions, retrospective think-aloud sessions, and associated focus groups was transcribed verbatim into text format by a professional transcriber for subsequent thematic template analysis. Thematic analysis is a qualitative analysis method used for searching and identifying themes that occur within textual datasets [72]. Using this method enabled patterns in the dataset to be identified and categorized. Analysis of the semistructured interview data was both inductive as the development of the themes were data driven and deductive, beginning with predefined (a priori) themes that are theory driven and linked to the analytical interest of researchers [73]. The first stage involved creating a template that used the predefined codes specified by the TAM. Hence, analysis considered the participant perceptions of the 3D-MAP application in the context of the two high-level TAM themes: PU and PEOU, and themes that emerged in addition to these. Carrying out the analysis in this way conforms to what is considered to be a contextual constructivist approach to thematic analysis [74]. The entire dataset was then read and comments were assigned to the two predetermined TAM themes and other high level themes that emerged, moving similar texts into one place and rereading segments to ensure that connections were justified. The dataset was then examined iteratively through several stages of splicing, linking, deleting, and reassigning subthemes within each predetermined high-level theme. Subthemes in the context of individual participants’ accounts were considered, as well as examining the data across participants. Subthemes were included because of their relevance to the research question and not necessarily because of their prevalence across the data set, as is acceptable in qualitative research.

### Ethical Considerations

Ethical clearance was obtained from Brunel University research ethics committee before any data collection. Informed consent was therefore sought from each participant before taking part in the focus group interviews. Participants who took part in the study was assured of their confidentiality and anonymity and informed both verbally and in writing of the purpose of the study and of their right to withdraw at any time.

## Results

This section presents the results of an initial usability evaluation of the 3D-MAP prototype and the associated follow-up focus groups.

### System Usability Scale (SUS) Evaluation Results

The overall SUS results for 3D-MAP revealed a mean score of 65.8 (SD 16.05) on a 100-point scale. According to the SUS scoring matrix [68] this indicates that the application delivers “marginal-high” (acceptability range), “good” (descriptive adjectives), and “grade C” (curved grading scale) levels of usability. The results were analyzed with regards to the SUS usability and learnability subscales [65,71], which revealed scores that were significantly above the midpoint benchmark of 3.00: 4.02 (*P*=.004) and 4.27 (*P*=.001), respectively. This shows that participants were positive about the application’s usability and learnability. The Cronbach measure of consistency for the 2 constructs (0.67 and 0.63, respectively) achieved scores above the threshold of acceptable reliability of 0.6 for studies with small sample size [75]. A Spearman rho correlation was performed to determine the correlation between age and SUS scores. There was no significant correlation between age and SUS score (r=−0.041), which indicated that the 3D-MAP was considered usable independent of age. The study, therefore, continued with a follow-up analysis of the individual SUS items against the midpoint of 3.00, to identify any usability issues that the users in the sample experienced during the interactive task. To conduct this analysis, the negative SUS items (S2, S4, S6, S8, and S10) were reversed so that scores above 3.00 indicated a positive response. Table 2 presents a breakdown of the results of this analysis, accompanied by the full SUS item open-ended responses that participants provided.

Mean scores for all 10 SUS items, in absolute terms, were above the neutral midpoint of 3.00, which indicates that participants tended to be positive about the 3D-MAP application in terms of the SUS items. Furthermore, in terms of statistical significance, mean responses to all 10 SUS items were significantly higher than the midpoint benchmark. The results of the statistical comparison of the SUS scores and midpoint are now considered alongside the open-ended responses provided for each respective SUS item.

**Table 2:** Mean system usability scale (SUS) score and midpoint comparison.

SUS^a^ item	Midpoint	3D-MAP^b^, mean (SD)	Gap score	Df^c^	*t* test values	*P* value (2-tail)
S1: I think that I would like to use this 3D-MAP application frequently.	3.00	3.42 (1.062)	0.42	32	2.30	.03^d^
S2: I found the 3D-MAP application unnecessarily complex.^e^	3.00	4.09 (0.879)	1.09	32	7.13	<.001^d^
S3: I thought the 3D-MAP application was easy to use.	3.00	3.88 (1.083)	0.88	32	4.66	<.001^d^
S4: I think that I would need the support of a technical person to be able to use this 3D-MAP application.^e^	3.00	3.91 (1.234)	0.91	32	4.23	<.001^d^
S5: I found the various functions in this 3D-MAP application were well integrated.	3.00	3.94 (0.933)	0.94	32	5.78	<.001^d^
S6: I thought there was too much inconsistency in this 3D-MAP application.^e^	3.00	4.19 (0.873)	1.19	32	7.62	<.001^d^
S7: I would imagine that most people would learn to use this 3D-MAP application very quickly.	3.00	3.94 (1.435)	0.94	32	3.76	.001^d^
S8: I found the 3D-MAP application very awkward to use.^e^	3.00	4.26 (0.682)	1.26	32	10.28	<.001^d^
S9: I felt very confident using the 3D-MAP app.	3.00	3.82 (1.211)	0.82	32	3.88	<.001^d^
S10: I needed to learn a lot of things before I could get going with this 3D-MAP application.^e^	3.00	4.39 (0.747)	1.39	32	10.71	<.001^d^

^a^SUS: system usability scale.

^b^3D-MAP: 3D measurement aid prototype.

^c^Df: degrees of freedom.

^d^Indicates statistically significant ≥.05 confidence level.

^e^Responses of negative items were reversed to align with positive items, higher scores indicate positive responses.

Responses to item S1 indicated that participants tended to agree with the statement that they would like to use the 3D-MAP application frequently (mean=3.42, *P*=.03). However, when analyzing the open-ended responses to this item, it was apparent that some participants noted that they did not anticipate taking home measurements would be a task that they would have to carry out frequently. One participant disagreed with the notion of frequently using the application, as they reported having arm mobility issues that made using the handheld tablet device difficult.

Well hopefully we wouldn’t have to use it frequently, if we don’t need too many things. This is just mainly for ordering things to help us round the home isn’t it?

F14

I wouldn’t use the 3D app frequently because, well it’s hard to hold...but it’s easy to use.

F22

Participants tended to disagree with S2, that is, that they found the application unnecessarily complex (mean=4.09, *P*<.001). Participants did, however, highlight difficulties with rotating the 3D furniture models but felt that the other functionality of the application offered an easier way to record measurements compared with paper-based counterparts, as it did not require writing and that some of the other measurement arrows clearly showed exact areas to measure on furniture items.

Although I do find that rotation a bit of a pain...It’s not complex, you don’t have to do the writing and it gives you the arrows, it’s showing you where you have to measure across.

F3

Participants tended to agree that the application is easy to use (mean=3.88, *P*<.001). There were, however, usability issues expressed particularly relating to items that had multiple measurement entry arrows and in relation to rotating the 3D models using the touch gesture. One participant noted that the difficulties encountered were not associated with using the application or understanding the instructions given by it but rather, with the physical task of taking the actual measurements.

I think it needs some more development, but I would be happy to use it. I think the concept is really good...things like the toilet, when you’ve only got one measurement, you can get the link on that very quickly and easy. It’s when you’ve got multiple measurements to do the screen doesn’t seem...sensitive to what you need.

F3

It’s better than it would be because you’ve got clear arrows and everything to show you where you’ve got to measure. My problem is, if like me and a couple of other people, who live on their own and have, and are elderly, it’s hard to measure, it really is hard to measure.

F7

Responses to S4 indicated that participants did not feel that a technical person was needed to help them use the application (mean=3.91, *P*<.001). Nevertheless, some participants noted that they felt other user groups may require such assistance, depending upon factors such as age, functional abilities, and previous exposure to technology.

...it depends on age and whether you are, you know, you have like tablets. And it depends what your history is with you know with computer stuff.

F2

It depends very much on the individual person using it, but for me, no...it gives a bit of explanation for what you need. If you were using it with other patients it’s going to be a very wide range of abilities.

F21

There was a tendency to agree that the various functions of the application were well integrated (mean=3.94, *P*<.001). Some participants, however, commented that they had difficulty determining the measurement status of some items, that is, whether a measurement had already been entered or was still required.

It was integrated but I was hitting the screen avidly trying to get a measurement and it was already there but we couldn’t see it you know that sort of thing...a little measurement box.

F9

For S6, participants tended to disagree that there was too much inconsistency in the application (mean=4.19, *P*<.001). Nevertheless, some participants felt that the positioning of some of the measurement guidance arrows (particularly for the chair) could be further optimized and, in some cases, reported that the functionality appeared to be unresponsive.

It’s the responsiveness. It’s just that some of the arrows wasn’t responsive all the time.

F11

…the only one I would say I was a bit confused about was the chair,...it’s sort of measuring the depth of it, you know where the chair is but the arrow was underneath it.

F9

Participants tended to agree with S7 that most people could learn to use the application very quickly (mean=4.19, *P*=.001). One participant, however, considered that a step-by-step wizard type interface would be a useful design feature to reduce the amount of learning necessary to be able to use the interface and ensure that all measurements were collected as needed.

I think if they were taken through it bit by bit, like...a little icon to touch that says move to the next bit once you’ve answered the first bit.

F18

Responses to S8 tended to disagreed that the application was awkward to use (mean=4.26, *P*<.001). Some participants commented that the 3D models were easier to use and comprehend than their 2D counterparts. One participant reported issues with rotating the 3D models and suggested that on-screen rotation buttons may help this task.

It’s certainly better than having a picture.

F6

I had it back to front or upside down (the 3D-model). if it had...a little button with the arrows going four ways...you could turn your 3D thing round better than trying to do it with your fingers.

F30

Participants tended to agree with S9, that they felt confident using the application (mean=3.82, *P*<.001). However, one participant noted that their confidence could have been related to having used this with the study facilitator present, which would not be the case if it were used, as intended, independently within the home setting.

Well, because we’ve got someone with us, probably if we were doing it on our own, we’d be a little bit, ooh did I do that right, that sort of thing.

F23

The results for S10 show that participants tended to disagree that they had to learn a lot of things before they could start using the application (mean=4.39, *P*<.001) although the application demonstration provided at the start of the session was noted as being useful by one participant (F5).

### Semistructured Focus Group Discussion Results

**Figure 10:**
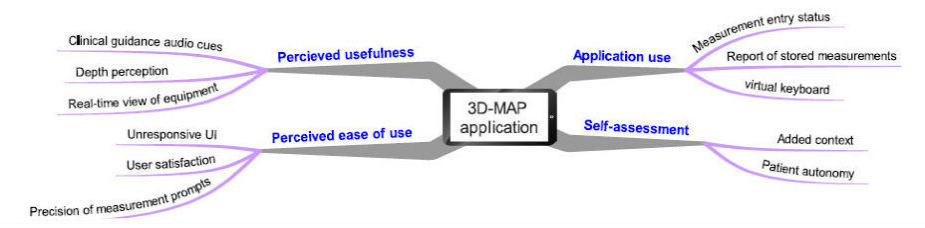
Thematic mind map of core themes and associated sub-themes.

Four high-level themes emerged as a result of the inductive and deductive thematic template analysis carried out on the data collected from the focus group discussion sessions. These themes were: (1) PU, (2) PEOU, (3) application use (AU), and (4) self-assessment (SA). An overview of the high-level themes and associated subthemes are presented in Figure 10.

#### Perceived Usefulness

Participants felt that the clinical guidance audio cues functionality was useful and made the 3D-MAP application easier to use. They commented that the audio cues provided useful instructions on how to take measurements and complemented the measurement arrows. However, some participants suggested that the 3D-MAP would have been even more straightforward to use if there were more audio cues to assist in the use of the application. Other participants also noted that the measurement arrows overlaid onto the 3D models were a useful aid in identifying the precise points that needed to be measured.

If there was the voice command throughout it would have been easier.

F8

Participants commented that the 3D models offered realistic representations of real-life items that were to be measured. They suggested that the 3D models afforded improved depth perception of the discreet points that should be measured for the task and improved the visual quality of the measurement guidance, compared with the paper-based equivalents. Other participants were enthusiastic about the capabilities that 3D visualization provides with respect to the clarity of the illustrations and differences between inner and outer length measurement arrows.

You need to have a diagram like this to show you the depth of the object...and the arrow showing you what you meant by the depth.

F5

Providing that 3D view so you know you can see where to measure...makes it more clear and distinguish whether to measure the inside length or the outside length.

F32

As an additional feature to enhance the usefulness of the application, there was discussion about adding some sort of augmented reality feature to the application that could deliver a real-time view of assistive equipment in place within the home. Participants felt that such a feature could help them to better understand what home adaptations (and items of assistive equipment) may look like when fitted and indeed what their function may be.

...whether you could input in the measurements of the room and where you ask to put something and then superimpose to see whether it would go and which best position for it in that particular space that you want to put it in.

F6

#### Perceived Ease of Use

Numerous issues were identified in relation to the usability of the application by this user cohort. Participants reported on the application’s unresponsive UI, particularly the difficulties that they experienced with some of the measurement arrows not responding to touch gestures. For example, clicking to insert measurement information for 3D models that contained multiple measurement arrows triggered a slow response by the application. Similarly, sluggish response times were noted when attempting to rotate 3D models that had multiple superimposed measurement arrows.

...the arrows for the measurements wasn’t always responsive. Some of them were fine, but the ones with say 4 you couldn’t input all of them. But the voice was very useful...

F3

In general, however, participants reported that they enjoyed using the application; to the extent that they expressed their interest of using it again within a home setting. Some participants elaborated on this point, suggesting that the application showed potential for use in practice, enabling patients to feel more involved in decisions and activities related to the provision of assistive equipment.

Actually I loved it. Because I’ve never measured anything before and put it in. so I’m not familiar with this measurement. So I loved it...I’ll go home and practice. I’ll get my Ipad out.

F7

I think it shows great potential for use in the field...and the patient would feel more involved. That is a good thing.

F9

One particular 3D model was regarded as being problematic in terms of providing ambiguous measurement guidance, that is, the 3D model of the chair. The measurement arrows for the chair and toilet were highlighted as a need for more precision if it is to have a much-desired effect in terms of users accurately interpreting the measurement guidance based on the use of precise visual prompts.

The chair, was I thought...more difficult, the width of the chair is it to the outer arms or it’s more likely that the seat position is...It’s that degree of precision.

F26

#### Application Use

Participants were positive about using this application in practice and were enthusiastic about using it as a guide to taking measurements within the home setting. However, in terms of current interface design features functionality, participants suggested some adaptations that they felt would improve the user interaction experience, and hence, the potential of using it in practice. Measurement entry status was identified as a feature that required improvement. Participants noted the importance of more clearly signposting when a measurement has been input successfully, for example, in addition to the current feature that superimposes the measurement onto the arrow once it has been input, it was suggested that a clear change in color of the input arrow would help to signify the measurement had been provided. An option to generate a report of stored measurements was also put forward as a valuable additional feature. It was foreseen that the benefits of such a feature included the service user being able to review all the measurements that had been provided but also the potential to enhance the level of dialogue between service users, clinicians, and assistive equipment providers after the measurements had been taken.

...the capacity to be able to save your measurements and refer to them you know once you’ve sat down with the practitioner or whatever so ok that’s what you’ve measured...and to be able to refer...back and look at it again and say are you sure you got the measurement right.

F30

Although participants reported being satisfied with the process of inputting measurements via the full Android virtual keyboard, some issues were raised about the type and size of the virtual keyboard. The launch of the full Android keyboard obstructed the view of the 3D model screen and consequently, suggested having a small numeric keyboard to enter measurements values was proposed. It was suggested that if a unit of measurement could be selected prior to inputting the values (ie, centimeters, millimeters, or inches) the full keyboard would no longer be necessary and could be replaced by a simple numeric keyboard.

*There’s a problem we’ve got here. When you have a touch screen or you touch the arrow you want and if you know you’re only going to put inches in or something like that, then you wouldn’t need to have a full keyboard...if you do you just need to have a small sort of standard dialler type touchscreen rather than the big one. But that’s how it suddenly occurred to me you know that the limitation of the device is probably causing some confusion there because it covers the screen when it comes up you see.* [F7]

#### Self-Assessment

The notion of patient autonomy was raised as a direct consequence of utilizing the 3D-MAP application. The process of enabling users to carry out self-assessment for the subsequent prescription and fitment equipment was seen as an opportunity to reduce the typical waiting time necessary for a clinician or technician to carry out the home assessment process and for the necessary items of equipment to be installed more rapidly. Indeed, the view shared by the majority of the participants was that deploying such an application in this way would be of benefit in this regard.

It’s the fact that you can do this yourself and you’re not waiting for somebody to say, ooh we can’t come until four weeks’ time to do the measurements for you, isn’t it (group agreement).

F11

Having this (the 3D-MAP application), you know, could help get equipment for the bed...stairs...handrails and obviously the chair...all the things that sort of promote independent living.

F28

Whereas participants were generally enthusiastic with respect to the concept of the 3D visualization approach to better interpret measurement guidance for the purpose of accurately gathering measurements, opportunities to extend the application’s functionality were also suggested. For example, recording or mapping the dimensions of the room and the other items therein were seen as a way of carrying out more in-depth falls risk assessments and hence may have added benefits in order to prevent extrinsic fall risks. Participants believed that the added context in which the furniture item is placed should be considered in conjunction with taking measurements of furniture items.

Rather than individual items, measuring height width and other things. If you had a bedroom...that would have been easier to see (all of the risks). Because then you could assess where your bed is and where your other furniture is...then you could think ways of other preventing falls.

F31

You’re measuring this and you’re measuring that. Surely you should measure the rooms...the places it’s got to go in. I mean a bath is fine so you measure that. Shouldn’t that be used in conjunction with something to do with room measurements.

F30

## Discussion

### Principal Findings

This study presented a novel mobile application that uses 3D visualization technology, designed to guide and assist older adult service users in the taking and recording of measurements as part of the assistive equipment provision process. A total of 33 older adults used the 3D-MAP application to engage in a measurement task of items of home furniture that are known to be associated with falls and are routinely measured as part of the AEPP. Based on the analysis of the quantitative SUS, data revealed that the sample of older adult participants attributed a score of 65.8/100 for its usability, which indicated that the application may be described as having “marginal-high” (acceptability range), “good” (descriptive adjectives), and “grade C” (curved grading scale) levels of usability. In terms of the two SUS subscales, OTs also tended to agree with statements relating to the usability and learnability of the application. The SUS results, therefore, indicate that there was agreement that the application was easy to use and that learning to use the application was also straightforward for this user cohort. However, despite some promising results and the outcome that the older adults who took part in this study were enthusiastic about the prospect of using the application within the home setting to carry out self-assessments, the findings indicate that there are improvements to be made to the application that may contribute to the successful adoption of such an application by an older adult cohort in practice. There was no significant correlation between age and SUS scores. However, this could perhaps be a consequence of the older adults in this sample being more familiar with using tablets or mobile phones, which may have mitigated any significant age-related effects. This also may explain why age was not found to be a factor involved in how users perceived the usability of the application. Analysis of the individual SUS items and associated open-ended comments, along with think-aloud and semistructured interview data provided detailed study outcomes relating to the perceived feasibility, usability, challenges, and opportunities of the application being deployed in practice. Table 3 presents a summary of the key study outcomes, and categorizes these in terms of the implications for deployment in practice and design and functionality considerations. Each outcome is mapped to its respective source, that is, the individual SUS item (S1-S10), and the high-level theme that emerged from the analysis of the semistructured interviews: PU, PEOU, AU, and SA.

**Table 3:** Study outcomes.

Areas of focus	Study outcomes	Source
**Implications for self-assessment in practice**			
	Confident using the application without assistance or supervision	S4, S9, S10
	Still some service user concerns about measuring furniture items independently	S3
	Valuable tool for self-assessment, patient involvement, and patient empowerment	SA, S1, PU^a^
	Sharing furniture measurements with clinicians	AU^b^
	Reduced time and resources overhead	SA^c^
	Provides an improved ability to visualize and understand measurement guidance	S8, PU
	Useful multimodal interaction features for clear measurement guidance	PU
	Indicate exact areas to be measured on furniture items	PU
**Design and functionality recommendations**			
	Provide usage instructions and short demo of key features	S4
	Develop improved 3D rotation function to improve visualization guidance	S4, PU, S3, S8
	Precise and unambiguous measurement arrow prompts for multiple measurements	S6, PEOU^d^
	Brighter visual interface	AU
	Provide context of the furniture items	SA
	Visualize equipment installations in real-time in context of the home	PU
	Provide smaller-sized numeric keyboard for measurement entry	AU

^a^PU: perceived usefulness.

^b^AU: application use.

^c^SA: self-assessment.

^d^PEOU: perceived ease of use.

In terms of the implications for self-assessment in practice, older adults reported that they felt comfortable using the application without any assistance or supervision (S3, S9, and S10). One participant reported to a feeling of apprehension with regards to the physical task of taking measurements on their own, in part due to advanced ageing factors (S3). However, a recent update of the health care act stipulates that “capacity must be assumed” for those responsible for carrying out ADL around the home and that patients must take ownership of their own care within reason, if they are capable of doing so [76]. Therefore, despite the development of applications such as 3D-MAP, designed specifically to provide enhanced levels of guidance and support (compared with traditional paper-based equivalents), there still appears to be some demand for more personalized support for some user types. The application was seen as a useful tool to promote independent living and to empower older adults to take ownership and be involved with parts of the AEPP (SA, S1, and PU). Some participants viewed the recording of measurements as being a valuable feature to have in order to send to clinicians as part of the equipment provision process, which could enable patients to take part in crucial aspects such as taking measurements of their home furniture (AU). This has potentially significant positive implications on the outcomes of current practice, particularly given that older adults who are empowered to participate in technology-assisted interventions are more likely to contribute to decisions made pertinent to them personally [77] which, in turn, could improve overall patient satisfaction, quality of life, and, ultimately, the level of engagement with assistive equipment [78]. There is also a potential time-saving advantage associated with this technology-assisted self-assessment approach, which means that patients can go ahead with assessments without having to wait for a clinician to conduct a home visit. Waiting times were also seen as another component in adopting the 3D-MAP application to facilitate self-assessment (SA). This is particularly advantageous given the growing demands on clinicians’ time, coupled with the increasing strain on publicly available health care resources [7]. Notably, participants remarked that they saw benefits of using 3D visualization, which they believe provides improvement in the depth perception required to improve the way in which the guidance is perceived (S8, PU). The application was perceived as a useful solution as compared with the existing 2D paper-based self-assessment tools. It provided a rich set of multimodal interaction features (ie, both visual and audio) to help interpret the measurement guidance and enable the recording of accurate furniture measurement (PU). Previous studies have shown that the combination of visual aids and audio features are both useful and effective in enhancing older users experience while interacting with software applications, particularly for those who have lower health literacy [79,80]. This is a promising and important outcome given that 50% of assistive equipment is abandoned by patients, partly due to inaccurate measurements being collected using the current 2D paper-based guidance [24]. Older adults viewed the application as a promising and practical tool, which they felt, enhanced the visualization of measurement guidance and helped to more accurately indicate the precise areas on furniture items that must be measured for the purpose of self-assessment (PU).

Several design and functionality recommendations emerged from this study, providing insights into how the application prototype could be further developed to align it with the needs of older adults if it is to be successfully suited to and adopted in practice by the intended user group. It was suggested that some users may require more detailed usage instructions and a short application demonstration (S4). This is in line with existing research focused on overcoming barriers to technology use and adoption by older adult users, which suggests that challenges often stem from lack of confidence as a consequence of being unfamiliar with some mobile technologies [81]. Other studies have found ways to assist older adults in addressing the lack of confidence is through adequate training, demonstrations, and providing built-in assisted features, which heightened competence and confidence levels when using technology [82]. Participants expressed experiencing difficulties while rotating some of the 3D models and found that the rotation controls were occasionally difficult to manage when they manipulated the perspective view of the 3D models (S4, PU, S3, and S8). This aspect of the functionality therefore requires further development, as it impacts the interpretation of measurement guidance. Participants commented on the need for clearer and unambiguous visual prompts to measure furniture items, as some prompts (particularly for the chair that has multiple inputs) seemed less clear and could compromise the reliability of older users effectively perceiving the guidance for accurate measurement entry (S6, PEOU). This requirement is particularly crucial given that the application was developed to enhance the visual quality of measurement guidance via the use of annotated 3D models to sufficiently locate end-to-end points on the measurement arrows. It was also commented upon that the interface needed brighter visuals as it impacts on one’s confidence and attitude while using the application (AU). Moreover, the current design of the arrows appeared to require more effort than expected to input measurements, which seems to impact participants’ level of confidence in using the application independently without support. Indeed, a body of research concerning the design and development of interfaces suited for older adults suggest a set of design guidelines for this particular older user cohort and infers that many usability issues can be addressed by adhering to those guidelines, whereas also assessing the effectiveness and efficiency of system functionality [60,83]. Other participants felt that measurements of the context in which the furniture item is located should be equally considered as gathering the dimensions of home furniture (SA). As an extension to this idea, one participant suggested a potential feature to visualize assistive equipment installations in real-time in the home before prescriptions are given (PU). Providing visual sense by overlaying virtual objects onto the real-world environment (in camera view), thus augmenting older users’ imagined changes to their home environment before it is physically adapted by AEPP, can decrease cognitive load, promote continuous engagement in health care interventions, and improve health outcomes [84]. Interestingly, there is evidence from a study investigating the application and effects of augmented reality in exercise interventions for fall prevention, which found an improvement on falls efficacy, gait, and balance [85]. Superimposing 3D models of assistive equipment within the home was viewed as having potential to increase the reality effects and participation during the intervention, giving patients the capability to visualize imagined changes to their home environment before it is physically adapted within the assistive equipment provision process. Participants expressed the need for smaller numeric keyboard style interface for measurement entry, as the full sized alphanumeric keyboard obscured the 3D model screen, which in turn could impact the integrity of the input and users forgetting what measurements they are inputting (AU). Previous research has shown older adults’ preference for onscreen numeric-style keyboards [86] and suggests that data entry should be kept to a minimum. The type of keyboard interface chosen should be relative to the amount of data entry activities performed by older adults [87]. There is also further evidence of onscreen numeric keyboard as the preferred interface for accurately recording numerical values and reducing the number of input errors in a health care setting [88,89].

### Limitations

Older adults recruited for this study were sourced primarily from active ageing exercise groups and hence, the sample in this study is likely to have been susceptible to selection bias. Furthermore, participants reported to be healthy, active, and familiar with the use of desktop computers, laptops, and mobile phones and also had some level of familiarity with touchscreen technology. Whereas this represents a skewed sample, it enabled the study to focus on evaluating the application and its functionality as opposed to the focus of perceptions being limited to basic usability issues that may arise from not having a basic understanding of the platform on which the application was deployed. Nevertheless, it is important to note that this sample may not be representative of the typical groups of older adults that OTs frequently engage with, and therefore should be taken into consideration when interpreting the results. The typical older adult patient profile is changing, as younger and more technologically aware generations make the transition into the older adult category, so the typical level of familiarity with ICT of this cohort will increase over time. Therefore, although the sample in this study is biased, such participants were recruited with the motivation of gaining insights from a sample, that may to some extent, better represent the more technologically aware older adult user group of the future. In relation to the TAM model, the deductive approach implemented in the analysis of qualitative datasets, via the two core TAM constructs, could be considered a limitation of this study. Adopting the thematic template approach in this study may have minimized the coverage of themes that would have emerged if a solely inductive approach was employed. Having said this, the approach enabled the analysis to be partly data driven, as well as focus in more detail on factors associated specifically with technology acceptance, which was in line with part of the aim of this study. Furthermore, it should be noted that no formal spot checks were carried out to ensure that participants adhered closely to the directions and guidance provided by the application. There is, therefore, a possibility that the lack of adherence observed when patients utilize paper-based guidance could similarly be a challenge to the tablet-based version of the guidance and something that should be taken into account when considering the results.

### Conclusions

This study investigated the experiences and views of 33 community-dwelling older adults who engaged in an interaction task with a custom built 3D-MAP application developed as a tool to engage in self-assessment tasks and assist them in taking and recording measurements as part of the AEPP. The usability of the 3D-MAP application was evaluated via the statistical analysis of participant responses to the SUS instrument. Perceptions regarding the feasibility, benefits, and challenges of using this application in practice were evaluated via the thematic analysis of individual interview and focus group discussions that were held after participants carrying out an interaction task. Based on the results, several implications for deployment of this application in practice were identified. Furthermore, numerous design and functionality recommendations were identified, which exemplify the interaction challenges that this cohort experienced with this 3D visualization technology. Overall, community dwelling older adults believed that the application delivered an improved visualization of the measurement guidance provided by traditional 2D paper-based guidance leaflets. The multimodal nature of the measurement guidance was also noted as a valuable benefit to deploying guidance via the mobile application. Furthermore, older adults were confident using the application without assistance and saw several benefits to deploying such an application in practice. Some of these included a perceived value in assisting with the self-assessment process, but also as a tool that could encourage patients to engage more fully in the delivery of their own care and collaboration with clinicians and associated decision making about their care. Further research is needed to establish whether such an application may be feasibly used by occupational therapists, family members, and regular care givers. It is also necessary to carry out further research to establish the clinical utility of this application in terms of the efficiency, effectiveness, and the relative accuracy and reliability of measurements that are recorded by older adults using the 3D-MAP application compared with 2D paper-based guidance leaflets. Furthermore, future research is needed to consider the use of an experimental design to empirically test the application against its 2D counterpart, to enhance and provide further insights into the findings presented here.

## Abbreviations

2D: two-dimensional
3D: three-dimensional
3D-MAP: 3D measurement aid prototype
ADL: activities of daily living
AEPP: assistive equipment provision process
AU: application use
ICT: information and communication technologies
NHS: National Health Service
OT: occupational therapist
PEOU: perceived ease of use
PU: perceived usefulness
SA: self-assessment
SUS: system usability scale
TAM: technology acceptance model
UI: user interface
